# Effect of Argan Oil on Lipid Production by *Yarrowia lipolytica* NRRL YB-423

**DOI:** 10.4014/jmb.2410.10052

**Published:** 2025-02-14

**Authors:** Rouna Alaaeldin, Bilge Sayin, Zerrin Polat, Mükerrem Kaya, Güzin Kaban

**Affiliations:** 1 Department of Food Engineering, Faculty of Agriculture, Atatürk University, Erzurum, Türkiye; 2 Department of Gastronomy and Culinary Arts, School of Tourism and Hotel Management, Ardahan University, Ardahan, Türkiye; 3 MK Consulting, Ata Teknokent, Erzurum, Türkiye

**Keywords:** *Yarrowia lipolytica*, argan oil, oleaginous, microbial oil, single-cell oil

## Abstract

The aim of this study was to investigate the effects of different concentrations of traditional, industrial, and cosmetic argan oils on lipid production by *Yarrowia lipolytica* NRRL YB-423 in a glucose-based medium. This study also explored the influence of different nitrogen concentrations on lipid and biomass production. Traditional argan oil had the highest oleic acid amount, whereas industrial and cosmetic argan oils had a higher linoleic acid amount. A lipid accumulation of 4.18 g/l was achieved with industrial argan oil, equivalent to approximately 65% lipid yield based on the dry cell weight. In addition, the results indicated that higher concentrations of argan oil led to increased lipid production. Correlation analysis showed that the addition of argan oil caused a change in fatty acid composition and an increase in linoleic acid amount. Linoleic acid increased in the presence of cosmetic argan oil (0.5 ml). The same effect was observed in the presence of 2 ml of traditional or industrial argan oil. In addition, when the amount of additional nitrogen was increased to 1 g/l, oleic acid amount increased in the control group. The nitrogen concentration used along with the argan oil type also caused changes in the correlations. The industrial argan oil group differed from the other groups in the presence of 1 g/l N. On the contrary, in the presence of an additional 0.5 g/l N, the industrial and traditional argan oil groups were closely correlated with each other.

## Introduction

Oleaginous microorganisms produce lipids that account for more than 20-25% of their dry weight [1]. Under specific cultivation conditions, certain oleaginous strains can synthesize lipids up to 70% w/w dry cell weight [2]. Yeast species utilized for microbial oil production include *Rhodotorula*, *Yarrowia*, *Candida*, *Cryptococcus*, *Lypomyces*, *Rhodosporidium*, *Mortierella*, and *Mucor*. *Yarrowia lipolytica* is considered a model microorganism for lipid synthesis because of its heterotrophic nature and capability to generate multiple products concurrently [3]. *Y. lipolytica* distinguishes itself from other oleaginous yeasts by its high productivity, ease of growth, ability to thrive in a broad pH (2.5 to 9.0) and temperature range (18 to 32°C), and utilization of various substrates such as unrefined raw materials and industrial wastes, and tolerance to salt and metal ions [4].

Hydrophobic substrates such as vegetable oils, fatty esters, crude oils, soaps, and hydrocarbons are preferred for microbial oil production. Additionally, oleaginous yeasts accumulate triacylglycerols rich in polyunsaturated fatty acids [5]. Factors influencing microbial oil production include the carbon/nitrogen (C/N) ratio, carbon and nitrogen sources, minerals such as sulfur, zinc, phosphorus, and vitamins (especially thiamine) in the culture medium, dissolved oxygen concentration, pH, incubation, and temperature. Moreover, the formation of secondary metabolites such as citrate may affect production [6]. The formation of microbial oils by oleaginous microorganisms occurs during secondary metabolic growth with nitrogen restriction and high carbon concentrations in the medium. When nitrogen is depleted in the medium, microorganisms convert the remaining carbon source into oil [7]. Nitrogen limitation is a controllable process parameter that positively influences lipid accumulation [8].

Different vegetable oil sources, such as waste cooking oil [9, 10], borage oil, sesame oil, rapeseed oil, echium oil, linseed oil [11], olive mill wastewater [12-14], olive oil [15, 16], canola oil [17], and rapeseed oil [18], have been used in several studies on lipid production by *Y. lipolytica*. Argan oil, an important vegetable oil derived from the Argan tree (*Argania spinosa* (L.) *Skeels*) is exported from Morocco [19] and distributed to various parts of the world by European and North American companies. It is rich in oleic and linoleic acids, unsaturated fatty acids, and natural antioxidants [20]. Argan oil, also known as "virgin oil," can be divided into different types based on the method used to process the argan oil kernels [21]. However, there is no information regarding the use of argan oil for lipid production. Therefore, this study aimed to investigate biomass and lipid accumulation of *Y. lipolytica* NRRL YB-423 using different argan oil types (traditional, industrial, and cosmetic) at different amounts (0.5, 1.0, and 2 ml), and with additional nitrogen sources (0.5 and 1.0 g/l) in the culture medium.

## Materials and Methods

### Microorganism and Culture Conditions

*Yarrowia lipolytica* NRRL YB-423 (ATCC 18942) was obtained from the American Type Culture Collection (ATCC, USA). The strain was preserved in cryovials containing Malt Extract Broth (MEB) supplemented with sterile glycerol at a concentration of 50% (w/v) and stored at -80°C. All argan oils used in this study were provided by Jibal Azyar Co., a commercial company based in Agadir, Morocco.

Lipid productions were conducted using culture medium with the following composition (g/l): glucose 30, yeast extract 0.50, KH_2_PO_4_ 7, Na_2_HPO_4_ 2.5, CaCl_2_·2H_2_O 0.15, (NH_4_)_2_SO_4_, 0.5/1.00; MnSO_4_·H_2_O 0.06, ZnSO_4_·7H_2_O 0.02, FeCl_3_·6H_2_O 0.15, and MgSO_4_·7H_2_O 1.50 [11]. The argan oils added to the medium included traditional, cosmetic, and commercial argan oils. Shake-flask experiments were conducted using 250 ml Erlenmeyer flasks filled with 50 ml of the culture medium, supplemented with 0.5, 1, and 2 ml of argan oil. Moreover, (NH_4_)_2_SO_4_ was used as an additional nitrogen source as 0.5 and 1.0 g/l. The culture medium was sterilized by autoclaving at 121°C for 15 min. The flasks were inoculated with 1 ml of exponential pre-culture containing 10^7^ cells. Following inoculation, flasks were incubated at 28°C and 180 rpm in a rotary shaker (JSSI-100, JS Research, Republic of Korea) for 120 h.

Glucose, yeast extract, KH_2_PO_4_, CaCl_2_·2H_2_O, ZnSO_4_·7H_2_O, MnSO_4_·7H_2_O, BF3-methanol, hexane, chloroform, and malt extract broth were supplied by Merck (Germany). NH_4_Cl was purchased from ISOLAB (Turkey). Na_2_HPO_4_, FeCl_3_·6H_2_O, (NH_4_)_2_SO_4_, MgSO_4_·7H_2_O, NaOH, HCl, and glycerol were purchased from Sigma-Aldrich Chemical Company (Germany). Fatty acid methyl ester mix was obtained from Supelco (FAME-mix, 4-7801, USA).

### Determination of Biomass Concentration

The dry cell weight was used to calculate biomass concentration. The cultures were centrifuged (Thermo Fisher Scientific, MR^2^3I, Germany) at 16,000 ×*g* for 15 min at 10°C. The cell pellet was washed twice with distilled water, dried to a constant weight at 95°C, and then weighed [22].

### Total Lipid Extraction and Methylation

To obtain total intracellular lipids, 8 ml of 4 M HCl was added to the dry biomass and kept at 60°C for 2 h. The acid-hydrolyzed biomass was stirred in 16 ml of a chloroform/methanol mixture (1:1) for 2–3 h at room temperature. Then, centrifugation was performed at 5,000 rpm for 5 min at room temperature to separate the aqueous upper phase from the organic lower phase. Finally, the lipid-containing sub-phase was removed with a Pasteur pipette, and the solvents were evaporated at 40°C in a vacuum rotary evaporator (Buchi Heating Bath B-490, Switzerland)[23]. Lipid production was calculated as the weight (g) of lipids produced per liter of medium [24]. Lipid yield was expressed as the percentage of lipids in the dry cell biomass.

To prepare methyl esters of fatty acids, 1 g of sample was weighed into a tube, 1.5 ml of 2 M methanolic NaOH was added and capped under nitrogen gas. The samples were left at 80°C for 1 h for saponification, and then cooled. Subsequently, 2 ml of BF3 methanol was added, capped with nitrogen gas, and left for 30 min at 80°C. After cooling, hexane (1 ml) and deionized water (1 ml) were added. The tubes were vortexed and centrifuged for 10 min at 6,000 rpm. The upper layer was placed in a new tube containing Na_2_SO_4_. After the addition of 1 ml hexane, 2 ml of the upper layer was transferred to amber vials. The vials were capped under nitrogen and stored at -18°C until analysis [25].

### Determination of Fatty Acid Composition

Fatty acid composition was determined using gas chromatography (GC, Agilent 6890N Agilent Technologies, Germany) with a flame ionization detection (FID) system. Helium was used as the carrier gas and the flow rate was set to 1 ml/min. CP-Sil 88 (100 m × 250 μm × 0.20 μm) was used as the column. The oven temperature was increased from 100°C to 200°C at a rate of 3°C/min and from 200°C to 250°C at a rate of 4°C/min. The injection block and detector temperatures were 250 and 280°C, respectively. Fatty acid methyl ester mix was used as the standard (FAME mix 47885-U, Supelco, Germany).

### Statistical Analysis

Argan oil types (traditional, industrial and cosmetic argan oil), different levels of these oils (0.5, 1, and 2 ml) and additional nitrogen concentrations (0.5 and 1.0 g/l) were considered as factors for lipid production. The experiments were replicated three times and were carried out in a 3 × 3 × 2 according to a completely randomized factorial design. The data obtained were subjected to analysis of variance, and Duncan’s multiple range test was used to compare the means to determine the effect of the factors on lipid and biomass production. In addition, cluster analysis was performed using the chiplot program to determine the relationship between the factors and fatty acids (http://www.chiplot.online).

## Results and Discussion

### Fatty Acid Composition of Different Argan Oils

The fatty acid compositions of traditional, industrial, and cosmetic argan oils are presented in Table 1. Myristic acid (C14:0) and palmitic acid (C16:0) amounts in the different argan oils were not statistically significant (*p* > 0.05). In contrast, significant differences (*p* < 0.05) were observed in the levels of oleic acid (C18:1) and linoleic acid (C18:2). The traditional argan oil exhibited the highest oleic acid amount (50.00%) (Table 1). Oleic acid and linoleic acid are the two primary fatty acids in argan oil. Previous studies have reported oleic acid values ranging from 42% to 51% and linoleic acid values ranging from 29 to 37% in argan oils [26-30].

### Biomass and Lipid Production by *Y. lipolytica* NRRL YB-423

**Fig. 1:**
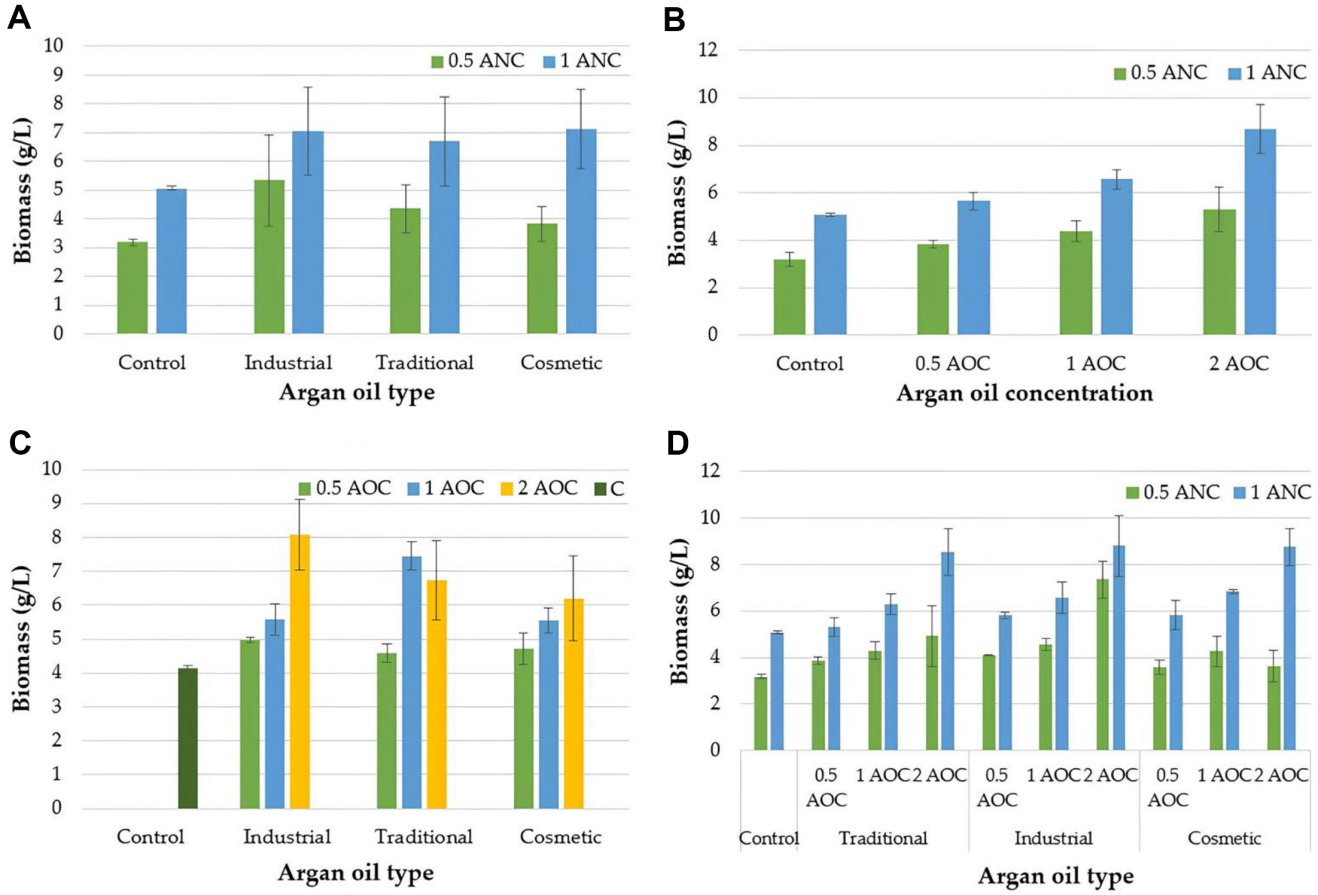
The effect of the interactions of ANC and AOT (A), ANC and AOC (B), AOT and AOC (C), ANC and AOT and AOC (D) on biomass concentration. (ANC: Additional nitrogen concentration, AOT: Argan oil type, AOC: Argan oil concentration, C: Control).

**Fig. 2:**
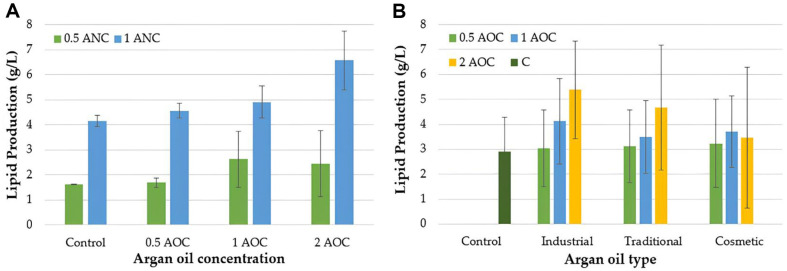
The effect of the interactions of ANC and AOC (A), AOT and AOC (B) on lipid production. (ANC: Additional nitrogen concentration, AOT: Argan oil type, AOC: Argan oil concentration, C: Control)

The result of biomass, lipid production, and lipid yield of *Y. lipolytica* NRRL YB-423 are presented in Table 2. Additional nitrogen concentration (ANC), argan oil type (AOT), and argan oil concentration (AOC) significantly influenced biomass concentration (*p* < 0.01). Increasing the nitrogen concentration led to an increase in biomass. Among the tested oils, industrial argan oil supported the highest biomass formation (6.20 ± 1.75 g/l). Also, the addition of 2 ml of argan oil to the culture medium resulted in the highest biomass concentration (7.00 ± 2.24 g/l). Conversely, the group without argan oil caused the lowest biomass (4.12 ± 1.03 g/l) (Table 2). Moreover, the ANC × AOT, ANC × AOC, and AOT × AOC interactions significantly affected biomass (*p* < 0.01). The use of argan oil and increased nitrogen concentrations caused an increase in biomass formation. Additionally, an increase in the nitrogen ratio led to increased biomass in both the control and groups treated with different types of argan oils. Argan oil caused the higher biomass formation when the additional nitrogen concentration was 1 g/l (Fig. 1A). Fig. 1B shows that biomass increased with increasing nitrogen and oil concentrations, reaching its highest value in the presence of 2 ml of oil and an additional nitrogen concentration of 1 g/l in the culture medium. The highest biomass was obtained by adding 2 ml of industrial argan oil to the culture medium. When cosmetic argan oil was used in the culture medium, the biomass increased, depending on the oil concentration. However, when traditional argan oil was used, the highest biomass was achieved at an oil amount of 1 ml (Fig. 1C). The highest biomass was obtained when 2 ml of argan oil was added to the culture medium containing 1 g/l of additional nitrogen in all the groups (Fig. 1D). Lipid production was significantly influenced by the addition of nitrogen and argan oil to the culture medium (*p* < 0.01) (Table 2). An increase in nitrogen concentration resulted in increased lipid production. Moreover, the addition of industrial argan oil resulted in the highest lipid concentration of 4.18 ± 1.91 g/l, corresponding to a lipid yield of approximately 65.02 ± 15.99% based on the dry cell weight. The highest lipid concentration of 4.50 ± 2.44 g/l was achieved when 2 ml of argan oil was added to the culture medium. Lipid production was also significantly affected by ANC × AOC and AOT × AOC interactions. The highest lipid production was observed when 2 ml of oil and 1 g/l of additional nitrogen were added to the culture medium (Fig. 2A). While lipid production increased in the groups using industrial and traditional argan oil in proportion to the amount of added oil, the highest lipid production in the group containing the industrial argan oil was observed when 2 ml of oil was added in the culture medium (Fig. 2B).

*Lipomyces starkeyi* [31, 32], *Rhodosporidium toruloides* [33, 34], and *Cryptococcus curvatus* [35] generally exhibit lipid accumulation ranging from 50 to 70% of the dry cell weight. *Y. lipolytica* was reported to produce lipids ranging from 30 to 50% [36-41] and 50 to 70 g/l [42]. Previous studies have employed metabolic strategies to enhance lipid accumulation of *Y. lipolytica* using glucose or glycerol-based media, resulting in 50-70% lipid content in shake flasks and bioreactor [43-46].

In a study investigating the effects of different oil sources (borage, canola, sesame, Echium, and trout oils) and oil industry residues (olive pomace oil, hazelnut oil press cake, and sunflower seed oil cake) on biomass and lipid accumulation of *Y. lipolytica* YB 423-12, the highest biomass (26.67 ± 0.09 g/l), lipid (16.45 ± 0.22 g/l), and lipid yield (61.67 ± 0.26%, g/g) were obtained with linseed oil [11]. Bellou *et al*. [47] examined the effect of nitrogen sources on lipid production by *Y. lipolytica* ACA-DC 50109 using (NH_4_)_2_SO_4_, NH_4_C_2_H_3_O_2_, yeast extract, tomato extract, meat peptone, and their combinations as nitrogen sources. In shake-flask cultivation, the highest lipid yield (14.5 ± 0.6%) was achieved using yeast extract as the sole nitrogen source. Another study conducted on *Y. lipolytica* investigated the effect of different concentrations of rapeseed oil in the culture medium and found that higher lipid (2.97 g/l) and lipid content (37.35% of dry weight) were obtained with a culture medium containing 40 g/l rapeseed oil than with 20 g/l [18]. Nitrogen limitation is a process parameter that positively influences lipid accumulation. During growth, the carbon flux is allocated between carbohydrates, lipids, nucleic acids, and proteins. Nitrogen is essential for protein and nucleic acid synthesis. Nitrogen limitation rapidly decreases the growth rate, whereas carbon assimilation rate gradually decreases. As a result, carbon flow shifts towards lipid synthesis, accumulating triacylglycerols [8]. Liu *et al*. [48] examined the effects of different concentrations (0 to 0.4 g/l) of added (NH_4_)_2_SO_4_ on citric acid and lipid production from waste cooking oil. Similar to our research, they observed a continuous increase in lipid production with increasing (NH_4_)_2_SO_4_ concentrations. Hence, the nitrogen concentration of the waste oil was insufficient for lipid production. Arslan and Taşar [49] utilized *Y. lipolytica* B9 as a producer strain for lipid production and found that increasing concentrations of (NH_4_)_2_SO_4_ (2-5 g/l) resulted in a continuous increase in cell biomass. When the production medium contained 50 g/l glycerol, the maximum lipid concentration (1.55 g/l) and lipid content (36.4%) were achieved in the presence of 3g/l (NH_4_)_2_SO_4_.

### Composition of Microbial Lipids Produced by *Y. lipolytica* NRRL YB-423

Table 3 presents the effects of additional nitrogen concentration, type and concentration of argan oil and their interactions, on the fatty acid composition. Palmitic (C_16:0_) and stearic (C_18:0_) acid amounts decreased with increasing nitrogen concentration. This factor did not affect palmitoleic (C_16:1_) and linoleic (C_18:2_) acid amounts. In addition, the highest values for the other fatty acids, except linoleic acid, were observed in the control group. As the amount of nitrogen increased, the amount of oleic acid (C_18:1_) increased, and a decrease with the effects of other factors was observed. The highest oleic acid amount was obtained 44.85 ± 13.07% when argan oil was not added to the culture medium. Unlike the fatty acids mentioned so far, the amount of linoleic acid was not affected by the additional nitrogen source factor but was significantly affected by the argan oil type and concentration (*p* < 0.01)(Table 3).

Regarding palmitic acid, no significant difference was observed between the nitrogen concentrations in the groups treated with traditional and industrial argan oil in the culture medium. An increase in the nitrogen concentration in the control and cosmetic argan oil groups resulted in a decrease in the amount of palmitic acid. The ANC × AOT interaction had a significant effect on palmitic acid amount (*p* < 0.01) (Fig. S1A). Also, the ANC × AOT × AOC interaction had a significant effect (*p* < 0.05). This interaction indicated that the highest amount of palmitic acid was presence in the control group in the presence of 0.5 g/l additional nitrogen, whereas the use of argan oil led to a decrease in this fatty acid (Fig. S1B).

Increasing the oil concentration led to a decrease in the amount of palmitoleic acid in the groups containing industrial argan oil. The highest palmitoleic acid amount determined in cosmetic argan oil was observed when 1 ml of oil was added to the culture medium (Fig. S2A). Additionally, in the group containing cosmetic argan oil, an additional nitrogen concentration of 1 g/l and oil addition of 0.5 and 1 ml increased the amount of palmitoleic acid. In the groups with traditional and industrial argan oil, an increase in the nitrogen concentration, except for the 0.5 ml oil concentration, led to an increase in the palmitoleic acid amount (Fig. S2B).

Increasing the nitrogen concentration caused a decrease in the amount of stearic acid in all the groups, including the control group. The highest amount of stearic acid was observed in the control group when an additional nitrogen concentration of 0.5 g/l was presence in the culture medium (Fig. S3A). All argan oil types resulted in lower stearic acid amounts than the control group, with the lowest amount determined in the group containing 2 ml of cosmetic argan oil (Fig. S3B). Simultaneously, the increased nitrogen concentration with different argan oil types and concentrations caused a decreased for stearic acid amount (Fig. S3C).

Argan oil types displayed differences depending on the added nitrogen concentration, with the highest oleic acid value determined in the control group with an additional nitrogen concentration of 1 g/l in the culture medium (Fig. S4A). The addition of 1 ml and 2 ml of oil did not significantly change the amount of oleic acid at the nitrogen concentrations tested. However, an increase in the nitrogen concentration in the control and containing 0.5 ml oil groups increased the oleic acid amount (Fig. S4B). Furthermore, the argan oil type caused variations in the amount of oleic acid, with a higher oleic acid amount achieved when 2 ml of industrial argan oil was added to the medium (Fig. S4C).

At both nitrogen concentrations used in this study, the control group exhibited lower linoleic acid values than the other groups. The highest linoleic acid value was obtained in the group containing cosmetic argan oil with an additional nitrogen concentration of 0.5 g/l (Fig. S5A). The addition of oil to the culture medium also increased the amount of linoleic acid, with the highest linoleic acid amount observed when 2 ml of argan oil was used in the presence of 1 g/l of additional nitrogen (Fig. S5B). Additionally, while the linoleic acid level increased with the increase in the oil added in the traditional and industrial argan oil groups, the highest linoleic acid amount was calculated in the group containing 0.5 ml of cosmetic argan oil (Fig. S5C). Moreover, the highest linoleic acid amount was determined in the group containing 2 ml of traditional argan oil with an additional nitrogen concentration of 1 g/l, and the cosmetic oil group containing 0.5 ml of argan oil with an additional nitrogen concentration of 0.5 g/l (Fig. S5D).

In another study using *Y. lipolytica* ACA-DC 50109 for lipid synthesis, the combination of technical glycerol and stearin in the medium yielded better outcomes than the combination of glucose and stearin. The lipids produced contained significant quantities of stearic acid and small amounts of palmitic, oleic, and linoleic acids. This resulted in a microbial oil composition resembling cocoa butter, indicating its potential for directing microbial metabolism [36]. Papanikolaou and Aggelis [50] developed a model to quantify the kinetic behavior of *Y. lipolytica* in terms of lipid accumulation and revealed that the highest intracellular lipid concentration and the maximum specific rate of lipid accumulation were achieved in media with elevated stearic acid content. In a study by Carsanba *et al*. [4], microbial lipids primarily composed of oleic, palmitic, and linoleic fatty acids were produced through the fermentation of *Y. lipolytica* H917 and *Y. lipolytica* Po1dL in a glucose-based, nitrogen-limited culture medium in shake flasks. Another study evaluated olive mill wastewater, a significant waste product of the olive oil industry, as a substrate. *Thamnidium elegans* and *Zygorhynchus moelleri* ensured cell mass production of 4.4 g/l and 3.5 g/l, respectively, in both surface and submerged cultures, with lipids accounting for approximately 60%(w/w) of the biomass. Similarly, oleic and palmitic acids were identified as major fatty acids [51].

### Results of Heat Map

**Fig. 3:**
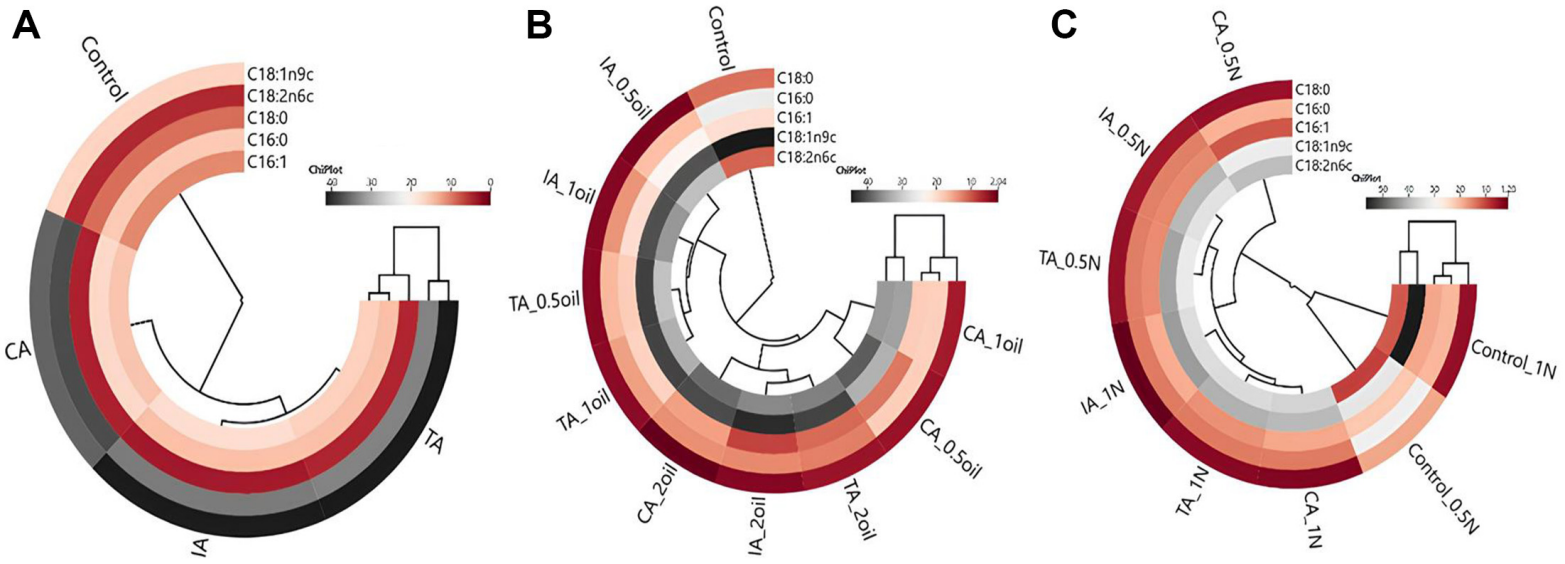
The cluster analysis of heat map showing the relationship between argan oil type and fatty acid composition (A) between argan oil concentration and fatty acid composition (B) and between additional nitrogen concentration and fatty acid composition (C). (TA: Traditional argan oil, IA: Industrial argan oil, CA: Cosmetic argan oil; argan oil concentration: 0.5oil, 1oil, 2oil; nitrogen concentration: 0.5N: 0.5 g/l, 1N: 1g/l).

Cluster analysis of the heat map showing the relationship between argan oil type and fatty acid composition (a), between argan oil concentration and fatty acid composition (b), and between additional nitrogen concentration and fatty acid composition (c) are shown in Fig. 3. In the correlation analysis based on the oil type factor, two main clusters were formed, and the control and argan oil groups were separated from each other (Fig. 3A). On the other hand, among the argan oils, cosmetic argan oil differed from both traditional and industrial argan oils (Fig. 3A). This result showed that the addition of argan oil caused a change in the fatty acid profile.

When the argan oil concentration was considered, two clusters were formed, and the control group was separated from all groups containing argan oil (Fig. 3B). The groups containing argan oil were divided into two groups, and both industrial and traditional argan oils were closely correlated with each other in the 0.5 ml and 1 ml oil groups. In the other group, it was observed that the groups containing 2 ml of argan oil types had close correlations, and it was revealed that this cluster was also closely related to the groups containing 0.5 and 1 ml of cosmetic argan oil (Fig. 3B). According to these results, the amount used for cosmetic argan oil can be reduced to 0.5 ml. This situation is important because cosmetic argan oil is more expensive than other types of oils owing to its high purity, specialized production processes, certification costs, and marketing.

In the correlation analysis conducted by considering the additional nitrogen concentration, two main clusters were formed (Fig. 3C). It was observed that the control group, which contained both 0.5 g/l and 1 g/l nitrogen, was in the same cluster and exhibited close correlation with each other and was separated from the groups containing argan oil. In addition, when the amount of additional nitrogen was increased to 1 g/l, oleic acid amount increased in the control group. On the other hand, the two subclusters formed within the second cluster separated the group in which 0.5 g/l nitrogen was used together with cosmetic argan oil from all other groups. The nitrogen concentration used along with the argan oil type also caused changes in the correlations. In the presence of 1 g/l N, the industrial argan oil group differed from the other groups. On the contrary, in the presence of additional of 0.5 g/l N, the industrial and traditional argan oil groups were closely correlated (Fig. 3C).

## Conclusion

In recent years, microbial oils have gained significant research attention because of their potential as alternative energy sources that can address both energy and environmental challenges. In this context, *Y. lipolytica* has emerged as a promising candidate for lipid production, utilizing a variety of cost-effective and different carbon sources. This study highlighted the potential use of argan oil as a substrate for lipid production by *Y. lipolytica* NRRL YB-423. Different types of argan oils and their concentrations, as well as the additional nitrogen concentration in the culture medium, affected the fatty acid composition. Additionally, argan oil caused an increase in linoleic acid amount in all groups compared with that in the control. As a result, it can be stated that certain groups of microorganisms such as *Yarrowia* may play a role in edible oil production and microbial oil production similar to the composition of expensive oils such as argan oil is thought to have the potential to contribute to both the food and cosmetic sectors.

## Supplemental Materials

Supplementary data for this paper are available on-line only at http://jmb.or.kr.



## Figures and Tables

**Table 1:** Fatty acid composition of traditional, industrial, and cosmetic argan oils (%).

Fatty acid composition	Argan oil type			Significance
	Traditional	Industrial	Cosmetic	
C_14:0_	0.18 ± 0.06^a^	0.34 ± 0.14^a^	0.18 ± 0.01^a^	ns
C_16:0_	18.61 ± 0.39^a^	20.02 ± 2.30^a^	21.65 ± 1.34^a^	ns
C_18:1n9c_	50.00 ± 0.25^a^	38.94 ± 4.43^b^	36.71 ± 5.47^b^	*
C_18:2n6c_	31.95 ± 1.67^b^	40.66 ± 2.07^a^	40.42 ± 5.02^a^	*

ns: not significant; any two means in the same column having the same letters in the same section are not significantly different at *p* > 0.05, **p < 0.01, *p < 0.05

**Table 2:** The effects of the additional nitrogen concentration, the type and concentration of argan oil on biomass, lipid production and lipid yield.

Factors		Biomass (g/l)	Lipid production (g/l)	Lipid yield (g/100 g dry cell)
Additional nitrogen concentration (ANC) (g/l)	0.5	4.38±1.23^b^	2.19±1.01^b^	50.62±14.45^b^
	1	6.78±1.48^a^	5.23±1.16^a^	77.71±8.99^a^
**Significance**		**	**	**
Argan oil type (AOT)	Control	4.12±1.03^c^	2.89±1.39^c^	66.59±17.29^a^
	Traditional	5.53±1.70^b^	3.75±1.88^ab^	65.40±20.43^a^
	Industrial	6.20±1.75^a^	4.18±1.91^a^	65.02±15.99^a^
	Cosmetic	5.48±1.99^b^	3.46±1.97^bc^	61.27±19.16^a^
**Significance**		**	*	ns
Argan oil concentration (AOC) (ml)	Control	4.12±1.03^d^	2.89±1.39^c^	66.59±17.29^a^
	0.5	4.74±0.99^c^	3.12±1.49^c^	62.68±20.04^a^
	1.0	5.47±1.20^b^	3.77±1.46^b^	66.09±18.36^a^
	2.0	7.00±2.24^a^	4.50±2.44^a^	62.91±17.45^a^
**Significance**		**	**	ns
**Interactions**				
ANC x AOT		**	ns	ns
ANC x AOC		**	**	ns
AOT x AOC		**	*	ns
ANC x AOT x AOC		*	ns	ns

ns: not significant; any two means in the same column having the same letters in the same section are not significantly different at *p* > 0.05, **p < 0.01, *p < 0.05

ANC: Additional nitrogen concentration; AOT: Argan oil type; AOC: Argan oil concentration

**Table 3:** The effects of the additional nitrogen concentration, the type and concentration of argan oil on fatty acid compositions of microbial lipids.

Fatty acid composition (%)						
**Factors**		C_16:0_	C_16:1_	C_18:0_	C_18:1n9c_	C_18:2n6c_
Additional nitrogen concentration (g/l)	0.5	17.98±6.18^a^	5.08±2.78^a^	6.96±4.30^a^	37.51±6.48^b^	32.45±8.88^a^
	1	15.18±2.40^b^	6.05±1.45^a^	3.25±2.06^b^	42.73±5.84^a^	32.76±8.04^a^
**Significance**		**	ns	**	**	ns
Argan oil type	Control	25.72±6.79^a^	6.70±3.60^a^	11.80±7.35^a^	44.85±13.07^a^	10.91±1.66^c^
	Traditional	14.76±2.08^c^	5.43±1.53^ab^	4.76±1.03^b^	41.55±3.42^b^	33.48±4.60^b^
	Industrial	14.87±2.32^c^	5.83±2.59^ab^	3.81±3.06^b^	41.43±3.49^b^	34.04±2.77^b^
	Cosmetic	17.08±4.73^b^	5.06±1.99^b^	4.50±2.58^b^	35.81±6.94^c^	37.53±4.50^a^
**Significance**		**	ns	ns	**	**
Argan oil con centration (ml)	Control	25.72±6.79^a^	6.70±3.60^a^	11.80±7.35^a^	44.85±13.07^a^	10.91±1.66^c^
	0.5	17.56±2.96^b^	6.03±2.40^a^	4.34±2.18^b^	38.17±6.65^c^	33.87±5.47^b^
	1.0	15.88±3.93^b^	6.40±1.38^a^	4.92±1.80^b^	38.65±4.81^c^	34.12±2.49^b^
	2.0	13.26±1.28^c^	3.88±1.30^b^	3.81±2.98^b^	41.96±4.29^b^	37.06±4.08^a^
**Significance**			**	ns	**	**
ANC x AOT		**	ns	**	**	**
ANC x AOC		ns	ns	ns	**	**
AOT x AOC		ns	**	**	ns	**
ANC x AOT x AOC		*	**	*	*	*

ns: not significant; any two means in the same column having the same letters in the same section are not significantly different at *p* > 0.05, **p < 0.01, *p < 0.05

ANC: Additional nitrogen concentration; AOT: Argan oil type; AOC: Argan oil concentration
