# Supplementary material for: Effect of Argan Oil on Lipid Production by Yarrowia lipolytica NRRL YB-423
Source: J Microbiol Biotechnol. 2025 Feb 14;35:e2410052. doi: 10.4014/jmb.2410.10052 (PMC11876015; doi:10.4014/jmb.2410.10052)
Supplement: Supplementary file 1 [file jmb-35-e2410052-supple.pdf]

J. Microbiol. Biotechnol. 2025. 35: e2410052
https://doi.org/10.4014/jmb.2410.10052

Supplementary Figures

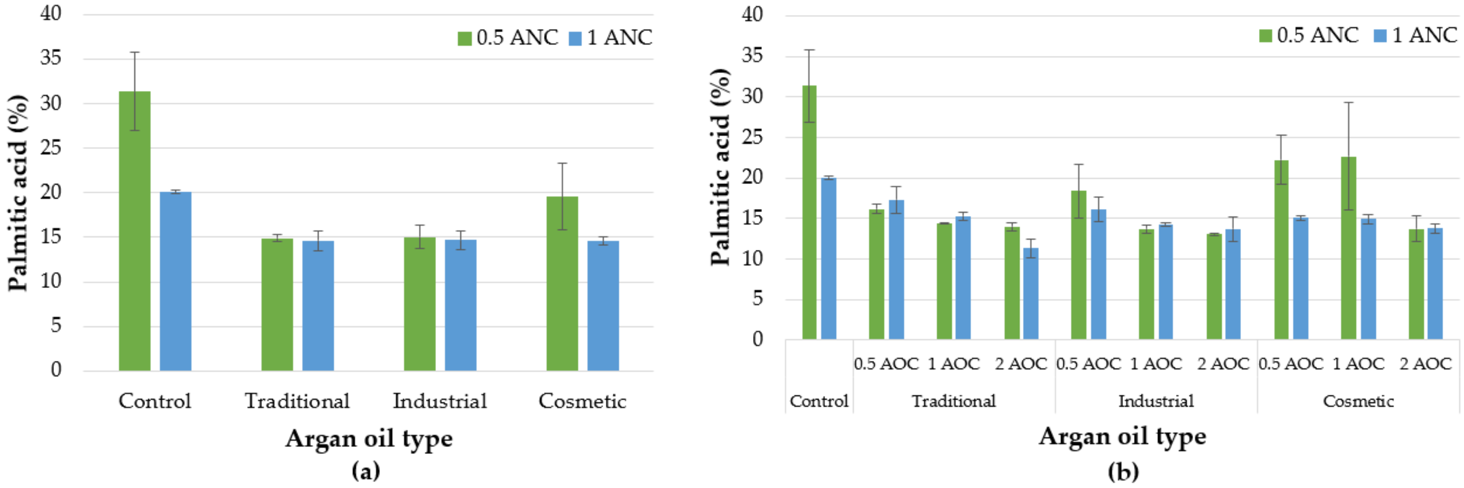

Fig. S1. The effect of the interactions of ANC x AOT (a), ANC x AOT x AOC (b) on palmitic acid amount (ANC: Additional nitrogen concentration, AOT: Argan oil type, AOC: Argan oil concentration)

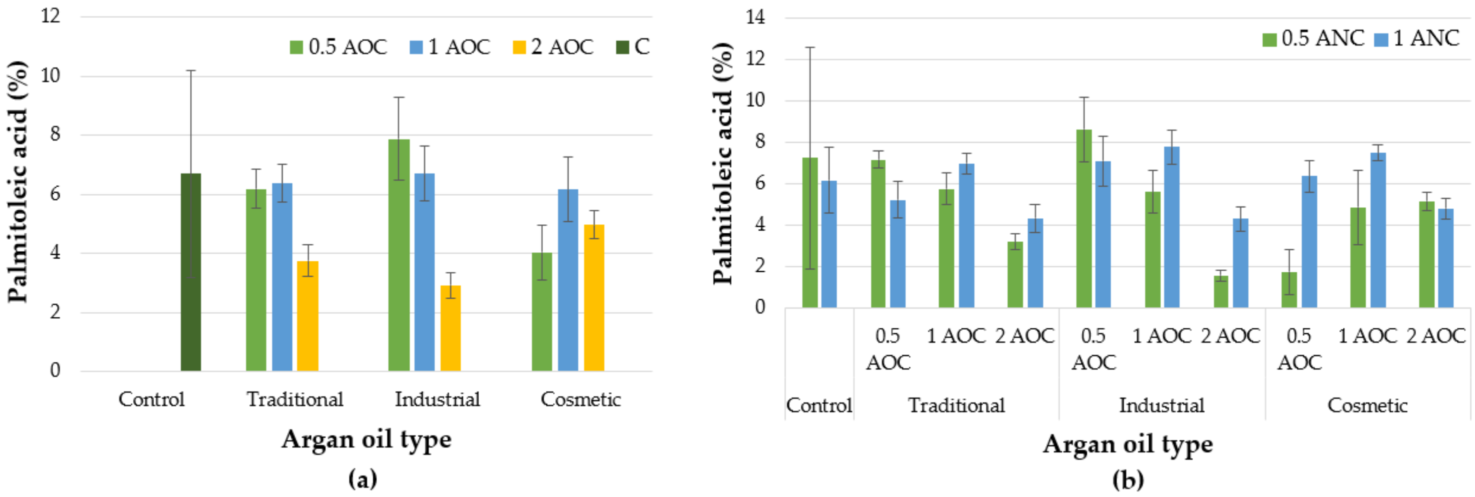

Fig. S2. The effect of the interactions of AOT x AOC (a), ANC x AOT x AOC (b) on palmitoleic acid amount (ANC: Additional nitrogen concentration, AOT: Argan oil type, AOC: Argan oil concentration, C: Control)

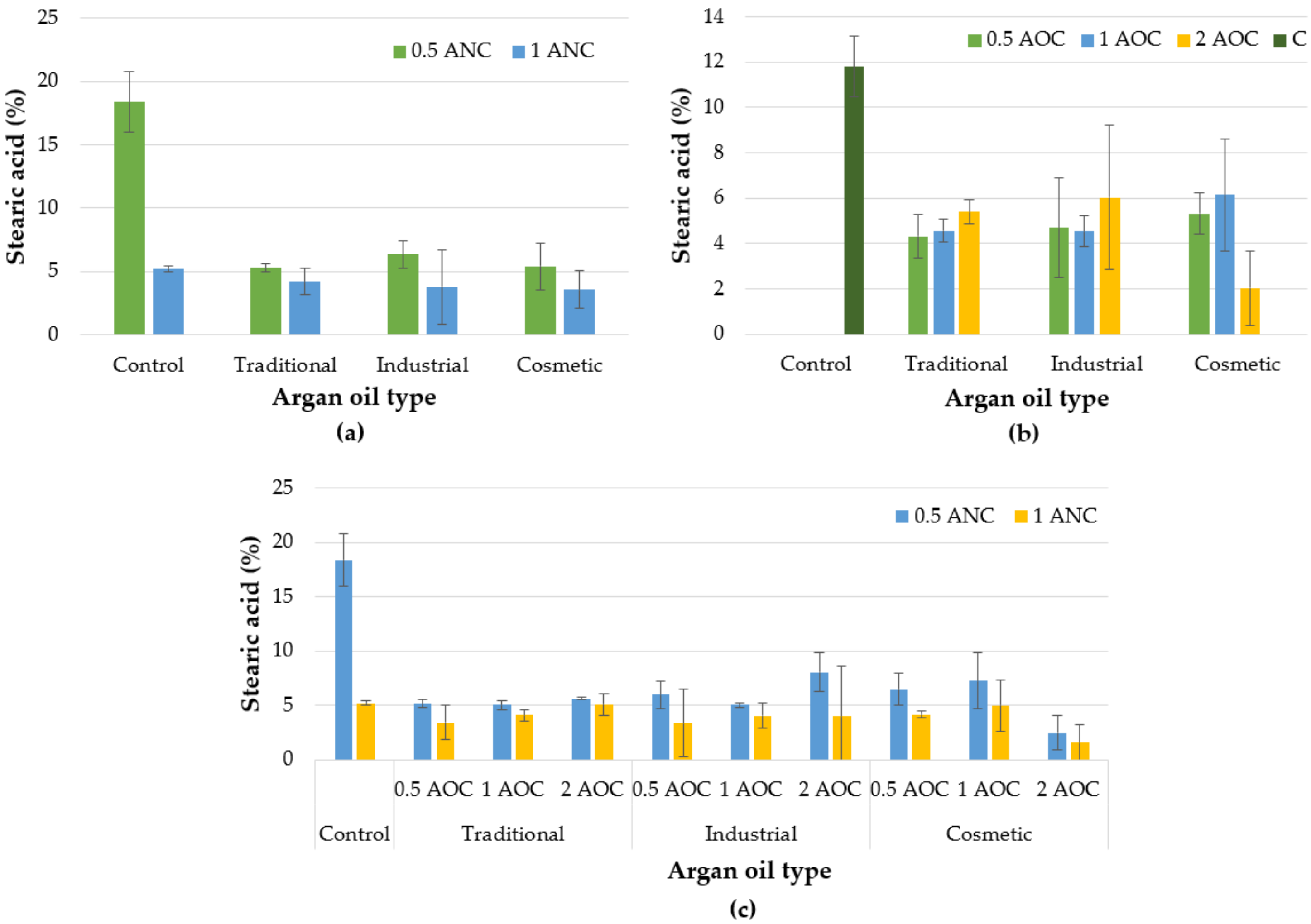

Fig. S3. The effect of the interactions of ANC x AOT (a), AOT x AOC (b), ANC x AOT x AOC (c) on stearic acid amount (ANC: Additional nitrogen concentration, AOT: Argan oil type, AOC: Argan oil concentration, C: Control)

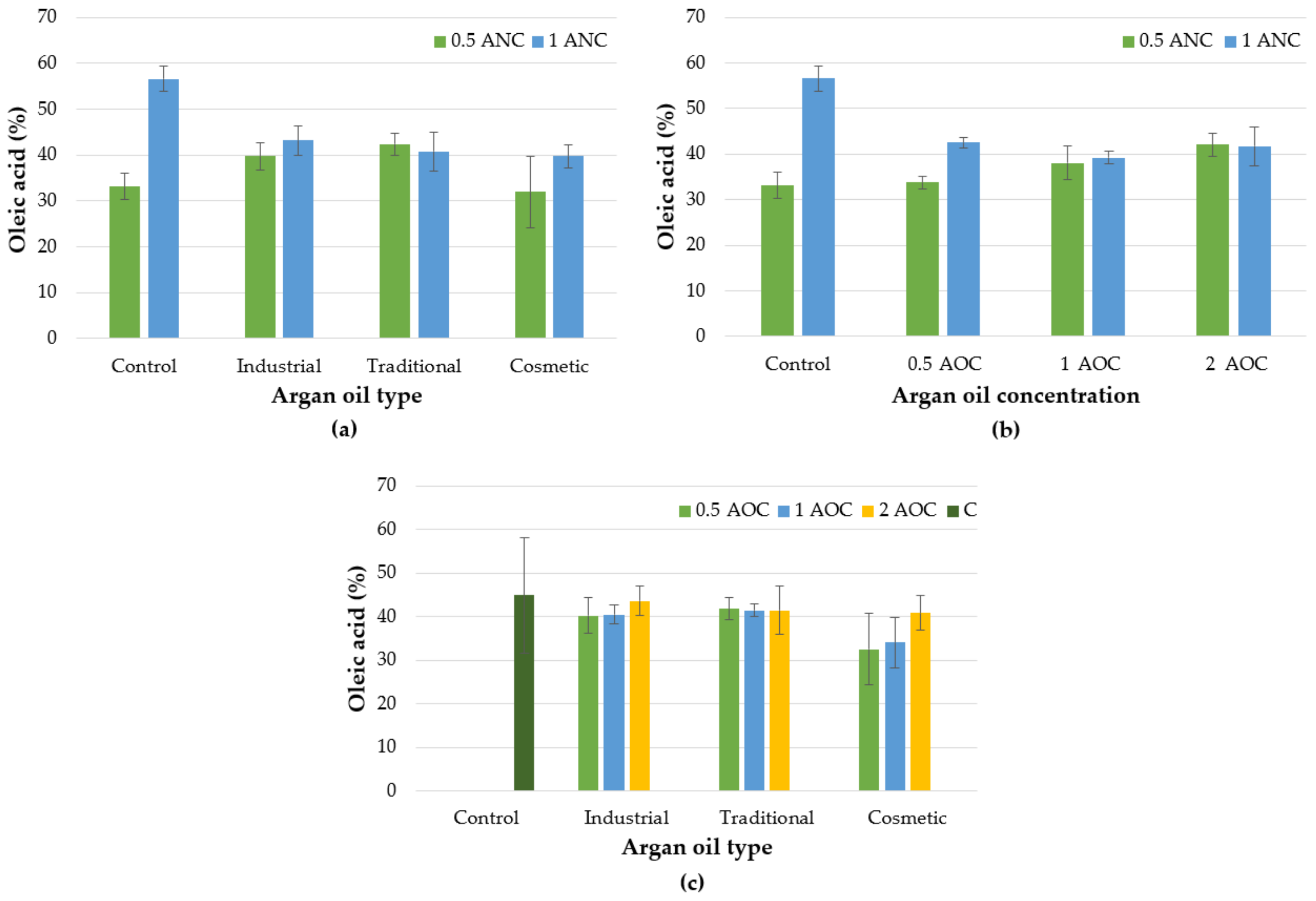

Fig. S4. The effect of the interactions of ANC x AOT (a), ANC x AOC (b), AOT x AOC (c) on oleic acid amount (ANC: Additional nitrogen concentration, AOT: Argan oil type, AOC: Argan oil concentration, C: Control)

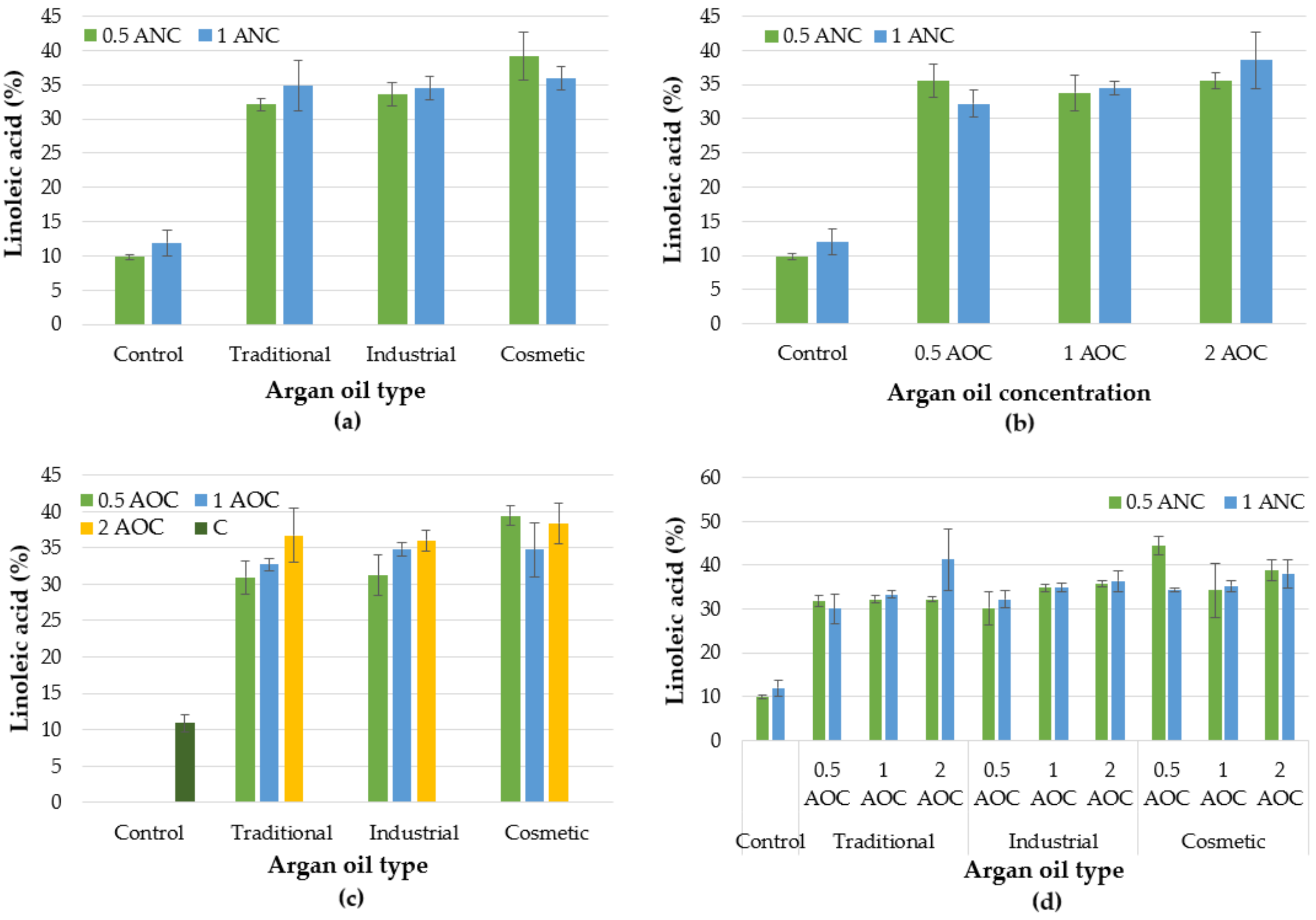

Fig. S5. The effect of the interactions of ANC x AOT (a), ANC x AOC (b), AOT x AOC (c) and (d) ANC x AOT x AOC on linoleic acid amount (ANC: Additional nitrogen concentration, AOT: Argan oil type, AOC: Argan oil concentration, C: Control)
